# Enhancing hospital safety: The impact of resilience on safety climate, safety performance, and occupational accidents

**DOI:** 10.1371/journal.pone.0328062

**Published:** 2025-07-14

**Authors:** Hamed Aghaei, Taleb Askaripoor, Morteza Siadat, Elahe Saleh

**Affiliations:** 1 Department of Occupational Health and Safety Engineering, TMS.C., Islamic Azad University, Tehran, Iran; 2 Department of Occupational Health Engineering and Safety, Damghan School of Public Health, Semnan University of Medical Sciences, Semnan, Iran; 3 MSC in Ergonomics, Semnan University of Medical Sciences, Semnan, Iran; 4 Social Determinants of Health Research Center, Semnan University of Medical Sciences, Semnan, Iran; Shahrood University of Medical Sciences, IRAN, ISLAMIC REPUBLIC OF

## Abstract

This study examined the impact of individual and organizational resilience on safety climate, safety performance, and the incidence of occupational accidents among hospital employees in Iran. The research aimed to determine whether resilience, at both individual and organizational levels, enhances safety climate and performance, thereby reducing occupational accidents. This cross-sectional study was conducted in 2024, involving 343 administrative and patient care staff from four government hospitals in Semnan, Iran. Validated questionnaires were used to collect the data. Structural equation modeling analyzed the relationships among the variables. The findings revealed that individual and organizational resilience positively influenced safety climate and performance, resulting in a reduction in occupational accidents. Personal competence emerged as the strongest indicator of individual resilience, while adaptive resilience significantly impacted organizational resilience. Safety climate was found to mediate the relationship between resilience and safety performance, underscoring its critical role in enhancing safety outcomes. This study emphasizes the importance of fostering both individual and organizational resilience to improve safety climate and performance in healthcare settings. Interventions focused on resilience and a positive safety climate are essential for reducing occupational accidents, especially in high-stress environments like hospitals.

## Introduction

Hospitals are among the most hazardous workplaces for occupational injuries and diseases. Employees face various hazards, including biological, physical, chemical, and ergonomic risks [1,2]. Gonçalves et al. [3] reported that hospital staff in the Spanish healthcare sector experience occupational accidents and illnesses at rates of 11.7% and 22.5%, respectively.

Evidence suggests that unsafe behaviors contribute to over 80% of workplace accidents [4,5] Unsafe behavior refers to any action that diverges from established safety standards, procedures, or instructions, thereby posing a risk to the workplace, the worker, or others [6]. In contrast, safety behavior adheres to safety guidelines. Safety performance is a broader concept than safety behavior, encompassing all activities by employees that contribute to promoting safety within the organization. Safety performance consists of two components: safety compliance and safety participation [7]. Safety performance is influenced by several factors, with safety climate being particularly important in reducing at-risk behaviors and improving safety outcomes [8,9]. Safety climate reflects workers’ understanding of an organization’s safety values and their shared perceptions of the importance of safety [10,11]. Previous research has shown that a positive safety climate promotes safety performance, human reliability, and reduces occupational accidents, making it a valuable indicator for assessing the effectiveness of safety plans and identifying changes in safety performance [12,13]. Liu et al. [14] demonstrated that safety behavior plays a crucial mediating role in the connection between safety climate and the occurrence of unintentional injuries. Safety climate is influenced by various factors such as management practices, psychological safety, leadership styles, and environmental conditions [15].

Some studies indicated that resilience influences safety climate [16,17]. Resilience is a multifaceted construct representing the ability of individuals or organizations to adapt, recover, and thrive in the face of adversity, stress, or challenges [18]. The importance of resilience in occupational safety and health is increasingly recognized as a key factor in workplace safety outcomes [19]. This variable fosters organizational capabilities to develop robust and adaptable processes for risk monitoring, model revision, and proactive resource allocation in response to adverse events [20]. Resilience encompasses both individual and organizational dimensions. Individual resilience involves successfully adapting to difficult experiences through mental, emotional, and behavioral flexibility [21]. Organizational resilience involves anticipating, preparing for, responding to, and adapting to changes and disruptions to ensure survival and success [22]. Hospitals and healthcare settings are complex socio-technical systems where resilience is crucial for effective patient care [23]. Enhancing individual and organizational resilience in these environments is vital for improving the safety climate, safety management, and overall safety outcomes, facilitating adaptation during challenging periods [24].

Hospitals, which face the dual challenges of patient care and a complex array of hazards, remain among the most dangerous environments for occupational injuries and diseases. To prevent accidents, it is essential to improve safety performance. This requires identifying, analyzing, and modifying the factors that contribute to safety performance. Despite the critical role of resilience in workplace safety, research on the effect of resilience on occupational accidents, particularly among hospital workers, remains limited. This study addresses this gap by investigating the simultaneous effect of individual and organizational resilience on safety climate, safety performance, and the incidence of occupational accidents among hospital employees in Iran.

Based on the literature review, the following hypotheses were formulated:

- * H*_1_: The relationship between individual resilience and occupational accidents is mediated by safety climate and safety performance.
- * H*_2_: The relationship between organizational resilience and occupational accidents is mediated by safety climate and safety performance.

**Fig 1:**
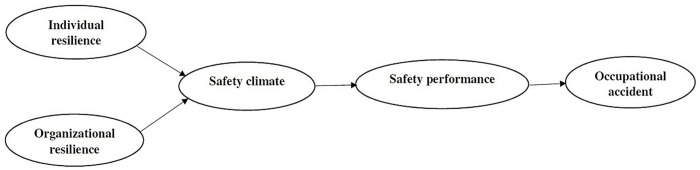
The conceptual model.

To test these hypotheses, structural equation modeling (SEM) will be employed to analyze the relationships among the variables. SEM is suitable for examining complex relationships and testing multiple hypotheses simultaneously. Fig 1 illustrates the study’s conceptual model, depicting the proposed relationships between individual and organizational resilience, safety climate, safety performance, and occupational accidents.

## Materials and methods

### Participants

The present study was conducted in 2024 with participants consisting of the administrative staff and patient care professionals from four government hospitals in Semnan, Iran. Hospital A had 472 staff members and 130 beds; Hospital B had 959 staff members and 273 beds; Hospital C had 335 staff members and 98 beds; and Hospital D had 171 staff members and 47 beds. All four hospitals have following department: emergency medicine, intensive care medicine, maternity, surgery, radiology, laboratory services, cardiology, nutrition and dietary services, social services, admission, dialysis, and decurity.

The number of participants was determined using the Morgan and Krejcie method [25]. With a total of 1,937 staff across the four hospitals, the calculated number of participants was 321. A multi-stage random cluster sampling method was employed for participant selection, with each hospital designated as a cluster. Within each hospital, the number of participants was allocated based on the size of each staff group, and participants were chosen randomly. All participants were informed about the study’s aims, and the questionnaires were completed by those who consented to participate. Inclusion criteria mandate hospital employment and a minimum of one year of experience at the current workplace, while exclusion criteria consist of unwillingness to participate or failure to meet these requirements. The recruitment period for this study ran from 5 May to 20 November 2024. The study protocol was approved by the ethics committee of Semnan University of Medical Sciences (ethics code: IR.SEMUMS.REC.1401.266). All study participants provided written informed consent and voluntarily participated in the research without any monetary compensation.

### Data gathering tools

During the present study, demographic data were collected using a checklist. Additional data were gathered through valid and standardized questionnaires.

Individual resilience was assessed using the Connor-Davidson Resilience Scale (CD-RISC), which evaluates five dimensions of resilience: personal competence, trust, positive acceptance, control, and spiritual influence, through 25 questions on a five-point Likert scale [26]. Mohammadi et al. [27]. evaluated the reliability and validity of the Persian version of this questionnaire, reporting a Cronbach’s alpha of 0.89, a content validity index (CVI) of 0.82, and a content validity ratio (CVR) of 0.99.

Organizational resilience was measured using a questionnaire developed by Prayag et al. [28], which assesses two dimensions of resilience, planned resilience and adaptive resilience, through 10 questions on a five-point Likert scale. Rastgar et al. [29] have confirmed the reliability and validity of the Persian version of this questionnaire, reporting a Cronbach’s alpha of 0.84 for planned resilience and 0.82 for adaptive resilience. Additionally, convergence analysis revealed minimum values for the composite reliability and average variance extracted (AVE) of 0.79 and 0.57, respectively.

Safety climate was evaluated using a Persian seven-item questionnaire specifically designed and validated by Ghasemi et al. [30]. The CVI, CVR, and AVE were utilized to evaluate the validity of the items, with the AVE calculated at 0.51. Furthermore, the Cronbach’s alpha coefficient for this questionnaire was reported as 0.87.

Safety performance was assessed with a Persian questionnaire designed by Ghasemi et al. [31], evaluating two dimensions: safety participation and safety compliance, through seven questions on a five-point Likert scale. The validity evaluation revealed that all questions achieved a CVR exceeding 0.99. Additionally, the reliability assessment demonstrated that Cronbach’s alpha coefficient was greater than 0.70. Additionally, employees were asked, “Have you had an occupational accident in the last 12 months?” to assess the incidence of occupational accidents.

### Data analysis

Demographic data were analyzed using descriptive statistics, while SEM was employed to test the study’s hypotheses. Data analysis was performed with IBM SPSS AMOS version 23.0 software. Absolute and comparative fit indices, including χ^2^/df, RMSEA, NFI, CFI, and TFI, were used to assess the goodness of fit of the conceptual model. The internal consistency of the data was evaluated by calculating Cronbach’s alpha coefficient.

## Results

This study involved 343 participants. Table 1 presents the general characteristics of the participants. The mean age and seniority of the participants were 37.1 years (±7.7) and 12.2 years (±7.2), respectively. The majority were female (67.1%), married (78.7%), had a bachelor’s degree (64.1%), and worked as patient care professionals (87.5%). In this study, all questionnaires showed Cronbach’s alpha coefficients greater than 0.8, indicating strong reliability.

**Table 1:** General characteristics of the participants.

Variable	Total sample (N = 343)
Age (year) – Mean (±SD)	37.1 (7.7)
Seniority (year) – Mean (±SD)	12.2 (7.2)
Gender – n (%)	
Male	113 (32.9)
Female	230 (67.1)
Marital status – n (%)	
Single	60 (17.5)
Married	270 (78.7)
Divorced or widowed	13 (3.8)
Education – n (%)	
Diploma	25 (7.3)
Postgraduate diploma	18 (5.2)
Bachelor’s degree	220 (64.1)
Master’s degree	53 (15.5)
Doctorate	27 (7.9)
Job – n (%)	
Nurse	171 (49.8)
Medical staff except nurse	94 (27.6)
Physician specialist	19 (5.5)
General physician	16 (4.6)
Administrative staff	43 (12.5)

Correlations between the study variables are presented in Table 2. Individual resilience was positively related to organizational resilience, safety climate, and safety performance (*p* < 0.01). Additionally, organizational resilience was positively related to safety climate and safety performance (*p* < 0.01). Furthermore, only safety climate and safety compliance had a significant negative correlation with occupational accidents.

**Table 2:** Correlations among dimensions of individual resilience, organizational resilience, safety performance, safety climate, and occupational accidents.

Variable	2	3	4	5	6	7	8	9	10	11
1. PC (IR)	0.63^**^	0.79^**^	0.69^**^	0.44^**^	0.28^**^	0.24^**^	0.24^**^	0.29^**^	0.39^**^	0.06
2. PA (IR)		0.67^**^	0.53^**^	0.45^**^	0.29^**^	0.28^**^	0.25^**^	0.25^**^	0.35^**^	−0.02
3. Tr (IR)			0.66^**^	0.46^**^	0.32^**^	0.29^**^	0.30^**^	0.26^**^	0.41^**^	−0.00
4. Co (IR)				0.37^**^	0.26^**^	0.28^**^	0.23^**^	0.49^**^	0.35^**^	0.06
5. SI (IR)					0.30^**^	0.26^**^	0.19^**^	0.22^**^	0.28^**^	−0.08
6. Pl (OR)						0.69^**^	0.52^**^	0.28^**^	0.42^**^	0.08
7. Ad (OR)							0.60^**^	0.35^**^	0.41^**^	0.10
8. SC								0.47^**^	0.50^**^	−0.18^*^
9. SCo (SP)									0.59^**^	−0.12^*^
10. SP (SP)										0.05
11. OA										

Note: IR: Individual Resilience; OR: Organizational Resilience; SP: Safety Performance; SC: Safety Climate; PC: Personal Competence; PA: Positive Acceptance; Tr: Trust; Co: Control; SI: Spiritual Influence; Pl: Planning; Ad: Adaptive; SCo: Safety Compliance; SP: Safety Participation; OA: Occupational Accident.

* p < 0.05;

** p < 0.01

Table 3 summarizes the fit indices of the model, providing both the calculated values and acceptable thresholds for each index. Given the reported fit indices, the structural model demonstrates a strong alignment with the data. All indices meet their established acceptable thresholds, indicating that the model effectively represents the underlying relationships in the data.

**Table 3:** Fit Indices of the structural model.

Fit Index	Value	Acceptable value
χ^2^/df	2.55	< 3
CFI	0.94	> 0.9
TLI	0.92	> 0.9
NFI	0.91	> 0.9
RMSEA	0.07	< 0.08

**Fig 2:**
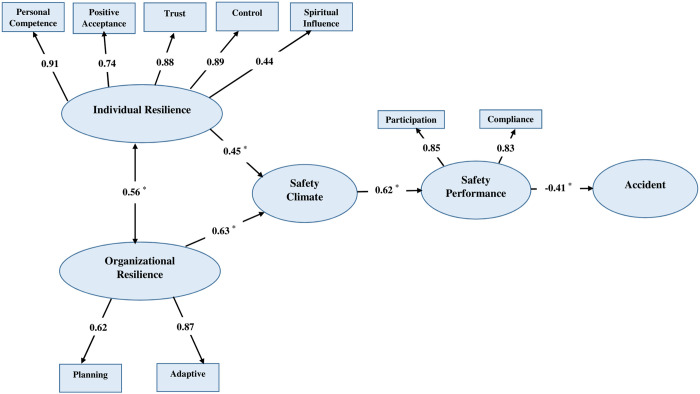
The study SEM model. * *p* < 0.001.

The findings from the hypothetical analysis, which evaluates the relationships between resilience, safety climate, occupational accidents, and safety performance, are illustrated in Fig 2. Among the five indicators of individual resilience, personal competence (indicator weight: 0.91) had the strongest effect, while spiritual influence (indicator weight: 0.44) had the weakest effect. Among the two indicators of organizational resilience, adaptive demonstrated a stronger impact compared to planning. Furthermore, participation (indicator weight: 0.85) and compliance (indicator weight: 0.83) had nearly identical impacts on safety performance.

Table 4 presents all information about each path. From this information and the model in Fig 2 it can be observed that the path coefficient between organizational resilience and individual resilience was 0.56, suggesting a statistically significant interrelationship and mutual reinforcement (*p* < 0.001). Additionally, the relationships between organizational resilience and safety climate, as well as individual resilience and safety climate, were also statistically significant (*p* < 0.001), with path coefficients of 0.63 and 0.45, respectively. Furthermore, safety climate exerted a significant effect on safety performance, with a path coefficient of 0.62. Safety performance also had the strongest negative impact on the occurrence of occupational accidents.

**Table 4:** Significance level of each path in the model.

Path						
From	To	Unstandardized path coefficient	Standardized path coefficient	*SE*	*t*	*p*
IR	Safety climate	0.48	0.45	0.09	5.33	^***^
OR	Safety climate	0.67	0.63	0.08	8.12	^***^
Safety climate	Safety performance	0.63	0.62	0.06	10.5	^***^
Safety performance	Accident	0.43-	-0.41	0.07	-6.14	^***^

*** *p* < 0.001

**Note: **IR: Individual Resilience; OR: Organizational Resilience

Finally, based on these results, *H*_1_ was accepted because safety climate and safety performance had mediating roles between individual resilience and occupational accidents. Also, *H*_2_ was accepted because the path between organizational resilience and occupational accidents was observed to be significant.

## Discussion

This study investigated the impact of individual and organizational resilience on occupational accident among employees in selected Iranian hospitals considering safety climate and safety performance as two mediating variables.

The results support both hypotheses, indicating that both individual and organizational resilience positively impact safety climate, which in turn enhances safety performance and decreases the incidence of occupational accidents. These findings are supported by relevant scientific literature and explore the broader context of resilience and safety in healthcare environments. Each of these factors is explained in the following sections.

### Individual resilience and safety outcomes

The findings of this study indicate that individual resilience has a positive impact on safety climate and safety performance. Consistent with these results, Britt et al. [32] reported that resilient individuals are more inclined to engage in proactive safety performance, such as reporting hazards and participating in safety training. These actions reinforce the importance of safety within organizations, thereby contributing to a stronger safety climate [32]. Additionally, research in the realm of information security has shown that resilience is linked to enhanced awareness and more secure behaviors, even in the face of job-related stress [33]. Chen et al. [34] further noted that resilient individuals foster a positive safety climate by modeling safety performance, supporting their colleagues, and advocating for safety improvements. However, these findings differ from those of Sadeghi Jozani et al. [35] reporting individual resilience cannot moderate the negative impact of trauma load on cognitive failures and human errors. These discrepancies can be attributed to the different occupational settings and safety outputs.

The present study focuses on Iranian hospitals is particularly significant, as healthcare workers in this region encounter unique challenges, including limited resources, high patient loads, and socio-political stressors. In this context, individual resilience may play an even more crucial role in upholding safety standards [36,37]. Additionally, several studies found a positive correlation between resilience and job satisfaction among Iranian nurses [38,39].

A proposed model elucidates how individual resilience positively influences safety outcomes by mediating the relationship between factors such as neuroticism, mindfulness, self-efficacy, coping strategies, and psychological adjustment [40]. Conversely, resilience is negatively correlated with psychological stress, as individuals with high resilience are better equipped to manage stress, maintain focus, and adhere to safety protocols, even in high-risk situations [41]. Evidence also suggests that resilient individuals are less likely to experience burnout, a common issue in healthcare settings that can adversely affect safety performance. Resilient individuals demonstrate superior capabilities in managing workplace stress and are less prone to burnout [42]. The mediating role of resilience in the relationship between job stress, burnout, sleep disturbances, job satisfaction, productivity, and safety outcomes highlights the need for resilience-focused training programs to enhance psychological well-being and safety performance in organizations [33,34]. For instance, Babanataj et al. [43] found that resilience training significantly reduces occupational stress among critical care nurses.

This study employed the CD-RISC to evaluate individual resilience, which is comprised of five dimensions: personal competence, trust, positive acceptance, control, and spiritual influence [26]. The findings revealed that personal competence exerted the strongest effect on individual resilience, whereas spiritual influence had the weakest effect. This supports prior research highlighting the critical role of personal competence in enhancing resilience and achieving positive outcomes. Competence, which encompasses self-efficacy and problem-solving skills, is a fundamental element of the resilience process [44]. Some studies have indicated that workplace spirituality positively affects individual resilience among hospital employees [45,46]. The discrepancy between the present study’s results and previous research, particularly regarding the weaker influence of spiritual factors, may reflect the secular nature of the workplace in the context of this study. While spirituality can be a significant source of resilience for some individuals or cultures, its influence may be diminished in professional environments dominated by secular values [47]. Moreover, there is a significant lack of research on the dimensions of resilience in workplace settings, including the spiritual variable [48]. Thus, additional studies are needed to fully understand the dimensions of individual resilience, especially in hospital environments.

### Organizational resilience and safety outcomes

The results of this study indicate that organizational resilience has a positive effect on safety climate and safety performance. Consistent with these findings, Ewertowski and Kuźmiński [49] reported that “safety management systems” and organizational resilience contribute to the safety climate. Additionally, another study found that organizational resilience had significant impacts on safety climate [16]. Notably, limited research has been conducted on the factors determining organizational resilience. Van den Berg et al. [50] reported that structural empowerment can enhance organizational resilience within the home care sector, provided there is a climate of psychological safety and strong commitment to empowerment from top management. Moreover, another studies indicated that to improve resilience and safety outcomes, organizations should focus on enhancing communication, engaging volunteers, and balancing centralized control with adaptive approaches [49,51].

The current study assessed organizational resilience using two dimensions: planned resilience and adaptive resilience. The findings revealed that adaptive resilience has a stronger impact on overall organizational resilience compared to planned resilience. This aligns with existing literature that underscores the significance of adaptability in fostering organizational resilience [52]. Adaptive resilience refers to an organization’s ability to respond flexibly to unforeseen challenges, while planned resilience involves pre-established strategies and protocols [53]. In healthcare settings, where uncertainty is a constant, adaptive resilience is particularly critical. For instance, during the COVID-19 pandemic, healthcare organizations with high adaptive capacity were better equipped to swiftly implement safety protocols, reorganize workflows, and support their employees, thereby minimizing disruptions to safety performance and maintaining high-quality care during this disruptive event [54]. This underscores the need for healthcare organizations to prioritize the development of adaptive resilience. This can be achieved by cultivating a culture of flexibility and innovation, encouraging employees to take initiative in addressing challenges, and providing resources that facilitate adaptive responses [55].

### The interplay between individual and organizational resilience

This study showed a significant interrelationship and mutual reinforcement between individual and organizational resilience. This finding is consistent with previous research indicating a positive correlation and mutual influence between these two constructs [56,57]. This dynamic interplay is particularly crucial in healthcare settings, where the inherent complexity and unpredictability of the work environment necessitate both individual and organizational resilience. For example, Lee et al. [58] found that nurses who perceived their organizations as resilient reported higher levels of individual resilience and greater engagement in safety performance. Seville [59] demonstrated that having strong team members and maximizing their potential, particularly during stressful times, is crucial for an organization’s resilience. Organizations can enhance resilience by creating a supportive work environment that encourages the development and application of resilience skills [60]. Therefore, based on the results of this study, it is suggested that improving safety outcomes in healthcare facilities and maintaining optimal performance, especially in critical situations, require planned interventions that simultaneously address individual and organizational resilience.

### Safety climate and its role in safety performance

This study found a significant positive relationship between safety climate and safety performance (path coefficient = 0.62). This result aligns with previous research demonstrating a consistent link between safety climate and improved outcomes, such as enhanced safety compliance, greater participation, better psychological well-being, and reduced accident rates [34,61,62]. Ghasemi et al. [31] demonstrated that a high level of safety climate can improve safety participation and safety compliance of nurses. Furthermore, Singer et al. [63] reported that a positive safety climate, particularly among frontline healthcare staff, is associated with a lower incidence of patient safety events. Similarly, Ghasemi et al. [64] demonstrated that safety climate can buffer the negative effect of problematic internet use on safety behavior. Therefore, the strong association between safety climate and safety performance underscores the importance of fostering a positive safety culture in healthcare settings, which can be achieved through strong leadership commitment, regular safety training, and open communication about safety concerns [65]. Notably, while evidence supports the positive effect of a strong safety climate on safety performance, existing methodological limitations emphasize the need for further research to definitively establish causality for the safety climate variable [66].

### The mediating role of safety climate

This study revealed that safety climate mediates the relationship between organizational resilience and safety performance, and between individual resilience and safety performance. This mediating role underscores the critical function of safety climate in translating resilience into improved safety outcomes. In essence, while individual and organizational resilience are important, their impact on safety performance is primarily channeled through the mediating effect of safety climate. This finding corroborates previous research identifying safety climate as a key mediator in the relationship between organizational factors and safety outcomes [13,66]. Moreover, another study found that safety climate significantly predicts affective organizational commitment, which, in turn, is associated with improved safety compliance and participation [67]. Resilience involves anticipating, preparing for, and recovering from adverse events, which can enhance the safety climate. Additionally, resilience significantly contributes to improving safety performance [17]. The current study extends this understanding by showing that safety climate also mediates the relationship between resilience and safety performance.

### Safety performance and occupational accidents

The present study revealed that safety performance has a negative significant impact on the incidence of occupational accidents, aligning with existing literature that correlates enhanced safety performance with lower accident rates [68,69]. Safety performance is comprised of two components: safety compliance and safety participation [70]. Our findings indicated that both dimensions exert nearly identical effects on safety performance, underscoring the equal importance of compliance and participation in mitigating occupational accidents. Supporting the results of present study, DeArmond et al. [70] found a negative correlation between safety compliance and participation and the occurrence of occupational injuries in the construction sector. Ghasemi et al. [31] concluded that training nurses was essential for enhancing safety compliance and participation, ultimately leading to a reduction in occupational accidents. Additionally, another study highlighted that effective safety management practices, including training and communication, contribute to accident reduction by enhancing safety compliance [71]. Furthermore, Wachter and Yorio [72] emphasized that worker engagement is vital in mediating the effects of safety management systems on safety outcomes, reinforcing the importance of engaging employees in accident prevention strategies. Based on the findings of the current study, it is recommended that interventions aimed at reducing occupational accidents should focus on enhancing safety compliance and participation while also fostering a positive safety climate.

The results of this study indicated that enhancing organizational resilience can improve the safety climate, ultimately leading to better safety performance and a reduction in occupational accidents. Moreover, enhancing individual resilience yields similar results to those of organizational resilience. Training programs focused on enhancing relaxation techniques, cognitive coping skills, and work-life balance can significantly boost individual resilience among workers [34].

### Limitations

This study, like others in the field, has several limitations. While it incorporated diverse job groups, factors such as cultural differences, variations in healthcare systems, and unique workforce characteristics may affect the applicability of the findings to other settings. Future research should examine these relationships in varied settings to validate our conclusions. Additionally, the cross-sectional design restricts causal inference; longitudinal studies are necessary to explore the temporal relationships among resilience, safety climate, safety performance, and occupational accidents. Finally, the emphasis on healthcare workers suggests caution in applying these findings to other industries. Future research should investigate the generalizability of these relationships across different sectors.

## Conclusion

This study emphasizes the importance of both individual and organizational resilience in influencing safety climate and performance, which in turn helps to reduce occupational accident rates in Iranian hospitals. Both types of resilience have a positive effect on safety climate, serving as a mediator in the relationship between resilience and safety performance. Individual resilience promotes proactive safety performance, while organizational resilience fosters a supportive environment that enhances adaptability to challenges. The findings underline the need for resilience-focused interventions to enhance safety outcomes in healthcare, particularly considering the unique challenges faced by hospital employees in Iran. Furthermore, nurturing a positive safety climate through strong leadership, transparent communication, and consistent training is essential for improving safety performance and reducing accident rates.
